# Metabolic Consequences of Thyroidectomy and Patient-Centered Management

**DOI:** 10.3390/jcm13237465

**Published:** 2024-12-08

**Authors:** Karina Wang, Seza A. Gulec

**Affiliations:** 1 Kiran C. Patel College of Osteopathic Medicine, Nova Southeastern University, Fort Lauderdale, FL 33328, USA; 2 Miami Cancer Research Center, Miami, FL 33181, USA; 3 HCA Aventura Hospital, Aventura, FL 33180, USA

**Keywords:** thyroid cancer, metabolic consequences, weight gain, thyroidectomy, thyroid hormones

## Abstract

Thyroidectomy has been post-operatively managed by hormone replacement therapy in order to satisfy the reference ranges of thyroid stimulating hormone (TSH) and thyroxine (T4) levels. While medication and standardized reference ranges have proven to be effective, many patients continue to report unintentional weight gain despite adequate amounts of treatment and levels of TSH and T4. Physicians, over the years, have become complacent to these “normal” ranges, and have ignored the metabolic consequences that are affecting thyroidectomy patients. This paper aims to redefine the approach to post-thyroidectomy clinical care by challenging the current standardized hormonal range values, exploring the gaps in thyroid hormone conversion, investigating the metabolic pathways of T3, considering the influence of inflammatory markers, and proposing the future for patient-centered management.

## 1. Introduction

Weight gain after thyroidectomy, despite seemingly adequate thyroid hormone replacement therapy, has been consistently reported among patients with both benign and malignant thyroid diseases. An estimated average weight gain of 2.13 kg (95% confidence interval; 0.95–3.30) was found across several studies with weight gain more prevalent in patients who underwent thyroidectomy for hyperthyroidism (5.19 kg) versus non-toxic goiters (1.55 kg) and malignancy (1.30 kg) [1]. Significant post-thyroidectomy weight gain in patients with thyrotoxicosis was found after the “normalization” of TSH, suggestive of pre-existing obesity with weight loss due to thyrotoxic activity [2]. In patients with Graves’ disease, weight gain was found to be greatest within the first three months of treatment [3]. Post-operative transient hypothyroidism has been a proposed theory to this phenomenon [2,4]. Similarly, euthyroid patients with non-toxic nodules experienced a more pronounced gain than in patients with autoimmune hypothyroidism early in their post-operative period, possibly due to goiters permanently reducing metabolic activity [5].

Levothyroxine (T4) therapy is the standardized treatment for thyroidectomy patients; however, weight gain has been noted despite adequate dosage and TSH values of within normal range. Comparative to healthy individuals with similar TSH levels, patients tend to have lower levels of free T3 and higher levels of free T4 after thyroidectomy [6]. This questions the adequacy of peripheral deiodination and the inability to compensate for the lack of endogenous T3 secretion. Even when TSH was maintained on levothyroxine treatment, patients experienced a hypothyroid state compared to their baseline [7]. The lower levels of free T3 and small variations in levothyroxine dosage could explain the significant decrease in basal metabolism rate and weight gain as a result [8].

Graves’ disease patients who underwent either thyroidectomy or radioactive iodine (RAI) treatment have a tendency to develop hypothyroidism and weight gain despite levothyroxine replacement [9]. This suggests that levothyroxine may lead to incomplete hormonal replacement. In addition, patients who underwent total thyroidectomy for thyroid cancer, even when they are on supraphysiological levothyroxine treatment (for TSH suppression) may complain of weight gain. Women in particular were shown to have a significant increase of 3.2% body weight during 3–5 years post operation despite suppressed TSH levels [10].

Overall, there are still many unknown factors regarding thyroidectomy, levothyroxine treatment, and the influence of either on patients’ individual physiology. Moreover, levothyroxine’s effect on the T3/T4 ratio is not well understood as there are no sensitive markers to assess the biological response of target organs and tissues [11]. There is still much need for research to understand the mechanisms behind these metabolic consequences. Specifically, it is crucial to redefine what hormonal values are considered “normal”, address the conversion capabilities of deiodinase, and investigate the mediators that follow T3 response. The contribution of inflammatory markers, such as preexisting high-sensitive C-reactive protein also deserves to be included in the discussion.

## 2. Methods

A PubMed and Google Scholar literature review was performed between July 2023 to November 2024. Search terms used were as follows: “thyroidectomy”, “metabolic consequences”, “levothyroxine”, “leptin”, “thyroid stimulating hormone”, “c-reactive protein”, “T3”, “T4”, “TSH”, “deiodinase”, “hypothyroidism”, “anti-thyroid peroxidase”, and “anti-thyroglobulin.” References from relevant articles were additionally reviewed.

## 3. Physiologic Thyroid Hormone Profile vs. Normal Laboratory Reports

The American Thyroid Association defines the normal range for TSH to be between 0.4 and 4.0 mlU/L [12]. However, other TSH ranges consist of values between 0.5 and 5.0 mlU/L [13]. The inconsistency of a normal TSH range is a topic covered at length in several research articles, further suggesting that patients may or may not have “normal” lab values depending on what standardized bell curve was used. It is important to note that bell curves are based on the 2.5th to 97.5th percentiles of values measured in the population tested, meaning that people could have normal TSH values despite being outside the set “normal” range [14]. While studies have been carried out to address the different range patterns for elderly, autoimmune, and pregnant patients, no studies have directly addressed the wide TSH range itself (a 10-fold difference between minimum and maximum values) [15]. This considerably large range raises the question of what exactly defines “normal”, and whether the current range for “normal” could truly be accurately applicable to all patients.

With small changes in free T4 resulting in large changes in TSH values, many physicians tend to address only TSH values with reflex T4 when warranted [16]. However, by having a large range applicable to all patients, physicians have become accustomed to accepting the current range values rather than considering the patient’s true physiological norm. In a study determining the optimal TSH ranges for reflex-free T4 testing, it was concluded that the majority of patients will have a TSH and free T4 within their respective normal ranges when screening for new thyroid disease. This made a diagnosis of a thyroid disorder more unlikely [16]. A patient’s individual physiology could also be influenced by lifestyle, age, sex, body mass index, environment, and genetics [17]. A thyroidectomy has the potential to disrupt a patient’s physiology, which may have adapted to the lifestyle and/or disease. This major change and disruption to a patient’s body has not been considered in how it would affect thyroid hormonal values.

**Figure 1:**
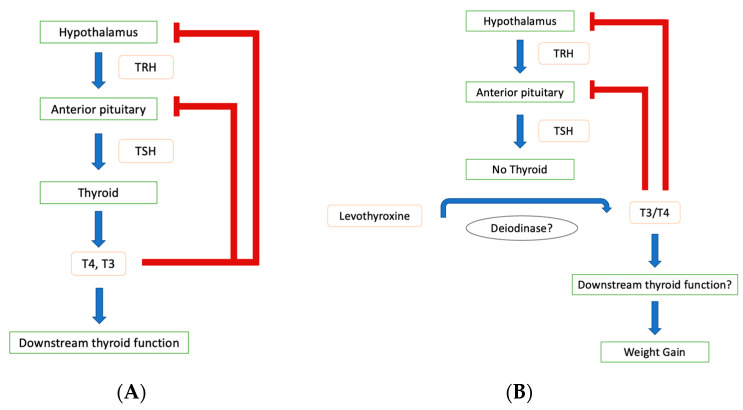
(**A**) Hypothalamus–pituitary–thyroid (HPT) axis. TRH: thyroid-released hormone secreted by hypothalamus. TSH: thyroid-secreted hormone secreted by anterior pituitary. Arrows indicate increased secretion. Red lines indicate negative feedback. (**B**) HPT axis depicted after thyroidectomy. This figure highlights potential factors that could cause potential weight gain. Levothyroxine acts as T4 hormone replacement and is converted to T3. Deiodinase and downstream thyroid functions remain unclear on how it can affect T3/T4 ratio and could serve as influence(s) for weight gain.

Treatment after the total thyroidectomy requires levothyroxine replacement; however, it has been shown that the reported normal hormone profiles cannot fully explain the unexpected weight gain that patients continue to face. While physicians claim euthyroidism solely based on the “normal” range values, patients have been consistently gaining weight despite having values within the range [1,2]. Moreover, the current conflicting data about post-thyroidectomy weight gain have all referenced the same values to determine whether a patient is “normal” rather than addressing the patient’s individualized physiology and hormone levels, both pre- and post-procedure. While the mechanism is unknown, it is proposed that the lack of endogenous production of T3 leads to a reduced T3/T4 ratio. While T4 is the major hormone secreted by the thyroid, T3 exerts the majority of the physiological effects (T4:T3 potency is around 1:4) [18]. Moreover, because T3 is the predominant inhibitor of TSH secretion and because low levels of T3/T4 increase TSH release, TSH secretion and its resultant values are sensitive to any minor changes in free T4 [19]. Research must consider a more individualized approach to thyroid hormone values by challenging the currently accepted range. Redefining normal could potentially further the understanding of the metabolic consequences of thyroidectomy (Figure 1A,B).

## 4. T4 to T3 Conversion

The conversion from T4 to T3 is determined by deiodinase. Type 2 deiodinase (DIO2) converts T4 to active T3 and is widely located in the brain, skeletal muscle, heart, and thyroid glands [20]. Physiologically, this enzyme takes T4 acquired from the bloodstream and converts it to T3 within the target tissue at a faster rate than Type 1 deiodinase (DIO1) [21]. DIO1, located mostly in the thyroid, liver, and kidneys, is responsible for catalyzing T4 to active T3 as well as T4 to inactive reverse T3 [22]. Type 3 deiodinase converts T4 into reverse T3 in the central nervous system and placenta [20,23]. Approximately 70–100 mcg of T4 and 30 mcg of T3 are, respectively, produced in a day. Roughly 25 mcg of T3 out of the 30 mcg are not produced by the thyroid [20]. Thus, eliminating the thyroid creates an unknown physiologic shift in T3 production and concern for T4 to T3 conversion [24].

In levothyroxine-treated patients, most circulating T3 (>80%) is due to the DIO2 conversion of T4 to active T3 [17,25]. The interaction between DIO2 and T4 ignites ubiquitination of DIO2—a molecular mechanism that modifies DIO2’s half-life through the binding of ubiquitin. This leads to the enzyme’s inactivation and degradation [26,27,28]. The decrease in T4 levels causes the extension of DIO2’s half-life, thereby increasing exothyroidal T3 production [17,29]. Through this phenomenon, DIO2 with levothyroxine treatment results in a normalized serum TSH but a reduced T3/T4 ratio.

Uniquely, DIO2 does not undergo ubiquitination and is more stable in the hypothalamus. Past research has shown that when a hypothyroid patient is treated with levothyroxine, the hypothalamus–pituitary DIO2 conversion suppresses thyroid-released hormone (TRH) and TSH. Because DIO2 is not ubiquitinated in the hypothalamus–pituitary, T3 production is greater here than in the periphery [30]. Therefore, TSH normalization occurs at a higher serum T4 level, one that is insufficient to normalize serum T3. Levothyroxine monotherapy has been proven to create a low serum T3/T4 ratio, meaning serum T4 levels are relatively high but inconsistency unclear with serum T3 levels. In treated patients, 15.2% had serum T3 below the normal reference range [6]. It has been noted that in order to normalize serum T3, levothyroxine dosage would need to increase, potentially leading to further TSH suppression [31,32,33].

The lack of thyroidal T3 production during levothyroxine therapy questions how reliable deiodinase conversion is, specifically under DIO2. Studies have shown that T3 deficiency could be the cause of weight gain; however, some argue that this deficiency would be subtle as serum T3 is generally normal during levothyroxine therapy [5,31,34]. Other sources have reported that levothyroxine does not guarantee true euthyroidism [6]. While mechanisms still remain unclear, the studies have proposed that different organ systems endure thyroid hormone deficiency differently depending on the presence of thyroid hormone receptors. For example, the thyroid hormone receptor alpha is predominately in cardiac muscles and thereby are better able to endure thyroid hormone deficiency [35,36]. The thyroid hormone receptor beta, found in adipose and other metabolic tissues, requires higher levels of T3 and does not normalize with levothyroxine therapy [37]. This could help explain why levothyroxine monotherapy can cause metabolic consequences such as weight gain. It also could explain why serum T3 levels could be considered normalized but the metabolism of T3 is not [17]. 

There are many unknowns regarding the DIO2 mechanism when the thyroid gland is not present. The consequences of thyroidectomy and how levothyroxine could influence the deiodinase enzymes remain uncertain. Moreover, the impact of thyroidal versus peripherally converted T3 is another topic that needs more research. While type 1 deiodinase is responsible for less than 20% of circulating T3 in levothyroxine patients, a defect in this pathway could also compromise the T3/T4 ratio. In order to reflect on how the T3/T4 ratio influences a patient’s metabolism, it is important to understand the mediators of T3 itself.

## 5. Effects of T4 on Intermediary Metabolism

In particular, thyroid hormone increases the basal metabolic rate by increasing the gene expression of Na+/K+ ATPase in different tissues. This leads to an overall increase in respiration rate, body temperature, and oxygen consumption [23]. Other proposed mechanisms include the uncoupling oxidative phosphorylation and direct modulation of thyroid hormone transporters and enzymes in the plasma membrane and mitochondria [11]. In metabolic studies on levothyroxine-treated patients with normal TSH levels, patients weighed about 10 pounds more and had slower BMR [38]. Another study comparing normal versus suppressed TSH levels in levothyroxine-treated women, the suppressed TSH level group had a mean TSH level of 0.14 mlU/L and a similar BMR to healthy controls. The normal TSH group averaged 2.1 mlU/L but had a significantly slower BMR [33].

Similarly, cholesterol metabolism is affected in levothyroxine-treated and low serum T3 patients due to low signaling in the liver and high serum cholesterol levels [25,39,40]. The normalization of serum cholesterol could be achieved by increasing the levothyroxine therapy dosage, leading to the normalization of serum T3 but a low TSH level outside of the reference range [41,42]. T3 is also known to stimulate the metabolism of carbohydrates by increasing glucose reabsorption, gluconeogenesis, glycogen synthesis, and glucose oxidation. It also plays a role in protein anabolism [23]. A decrease in T3 will inevitably affect both these systems as well.

## 6. Cellular Responsiveness to T3

While serum T3 measurements may be adequate in post-thyroidectomy patients, T3 may not be adequately utilized. The signaling pathways of T3 must be considered when addressing the metabolic consequences of thyroidectomies due to T3 having a higher affinity binding with nuclear receptors than T4. Currently, T3 is known to affect a variety of systems: brain, bones, cardiac, basal metabolic rate (BMR), blood sugar, and lipids [23]. With each system regulated by different deiodinase types, the post-thyroidectomy and/or levothyroxine treatments affect the T3/T4 ratio differently and, consequently, the signaling pathways of T3. 

Possible issues can arise in thyroid receptors as they are transcription factors already bound to DNA in the nucleus before ligand binding. These ligand-activated transcription factors bind to thyroid hormone response elements (TRE) of target genes, regulating gene expression by introducing epigenetic changes to influence RNA polymerase’s transcriptional efficiency on respected thyroid genes. Positively regulated target genes include fatty acid synthetase and growth hormone. Negatively regulated target genes include prolactin, TSH, and TRH [11]. Moreover, thyroid hormones affect a wide variety of cellular pathways and functions such as lipogenesis, insulin signaling, gluconeogenesis, and adenylate cyclase signaling [43,44]. It is important to note that thyroid hormones could also indirectly regulate through intermediate genes by binding to other transcription factors or by activating other cell signaling pathways. And while the hormones mainly act on transcription, they can affect mRNA stability, translational efficiency, and other levels of protein expression [11].

### 6.1. Signaling Pathways

Leptin, as an endocrine modulator, is secreted by fat cells to decrease appetite. Signaling by leptin to different areas of the hypothalamus is responsible for satiety or hunger. As a result, leptin indirectly stimulates the action of thyroid hormones and is regulated by T3. While there remains controversy as to the relationship between T3 and leptin, there is strong evidence for T3 in the upregulation of leptin gene transcription via the phosphatidylinositol 3-kinase (PI3K) pathway. Such activation would lead to adipostatic signaling to the brain. T4, on the other hand, has little to no effect on leptin mRNA [45,46]. In particular, the physiological dosage of T3 was shown to increase leptin gene expression in obese animals with diet restrictions [47]. Animals exposed to supraphysiological doses of T3 were subjected to a decrease in leptin mRNA [48].

Furthermore, the melanocortin pathway regulates and mediates leptin’s stimulation or inhibition on the thyroid axis via ligands on the arcuate nucleus of the hypothalamus [49,50]. It is suggested that alpha-MSH, a ligand that is induced by leptin, stimulates TRH release and increases TSH levels [49]. AgRP, a ligand suppressed by leptin, blocks TRH by antagonizing alpha-MSH [51,52]. Currently, it is unknown how leptin and the melanocortin pathways regulate under different physiological states, including thyroidectomy and levothyroxine treatment [50]. These pathways highlight potential mechanisms for weight gain in thyroidectomy patients. Further research into understanding how T3 mediates each pathway can clarify how a patient’s individualized physiology is affected.

11 beta-Hydroxysteroid dehydrogenase (11 beta-HSD), an enzyme expressed in adipose tissue that converts cortisol to its inactive form (cortisone), has also been shown to be influenced under T3 signaling by inhibiting 11 beta-HSD at both the liver and pituitary [53]. This signaling highlights another potential pathway that needs further study in post-thyroidectomy and treated patients as weight gain could be occurring due to increased active cortisol levels [54].

### 6.2. Genetic Predisposition

Metabolic abnormalities detected in levothyroxine-treated patients could be a result of the low serum T3 levels that could be compounded by other influences specific to a patient’s individualized physiology. Compromise or defects in deiodinase types could make the patient more sensitive to changes in target tissues [17]. Genetics also has the potential to predispose patients to obesity or insulin resistance. Depending on the ethnic background, a commonly inherited DIO2 polymorphism results in an amino acid substitution (Thr92Ala) in type 2 deiodinase [55]. This substitution of amino acids affects the ubiquitination and decreases the subcellular distribution of type 2 deiodinase [56]. In animals carrying the polymorphism, hypothyroidism was exhibited and associated with decreased physical activity and four times more sleep. In humans, Thr92Ala-DIO2 carriers have a transcriptional influence on central nervous system diseases. Mitochondrial dysfunction, oxidative stress, inflammation, and apoptosis were also noted [17]. Liothyronine therapy was shown to improve T3 brain signaling; however, levothyroxine had only partial improvement in Ala92-DIO2 patients with primary hypothyroidism [56]. Moreover, if Thr92Ala-DIO2 polymorphism carriers develop hypothyroidism and are subsequently treated with levothyroxine, serum T3 levels will decrease by 10% [17]. This decrease in T3 could potentially explain the metabolic imbalance occurring in thyroidectomy patients. In addition, other studies have potentially linked the relationship between Thr92Ala-DIO2 polymorphism and neuropsychiatric diseases such as recurrent depressive disorder [57]. With interactions between levothyroxine and fluoxetine (selective serotonin reuptake inhibitor) shown to lead to decreased levothyroxine serum levels and increased TSH levels, these patients who undergo thyroidectomies may experience further metabolic consequences due to complications of genetic predisposition and drug interactions [58]. It is important to note that there are dozens of proteins involved in the control of T3 signaling including membrane transporters, deiodinases, receptors, and their coregulators. This only emphasizes the potential for gene polymorphisms to interfere with the success of levothyroxine therapy post-thyroidectomy.

## 7. Inflammatory and Autoimmune States

Autoimmune thyroid diseases (AITD) are signified by the presence of anti-thyroid peroxidase (TPOAb), anti-thyroglobulin (TgAb), and thyroid-stimulating immunoglobulin (TSI) directed to TSH receptor (TSHR). It is important to note that while these antibodies may be present in generally asymptomatic and otherwise healthy individuals, their values are used to assess AITD patients and the status of their respective diseases [59,60]. A number of studies have shown that thyroid autoantibodies may be involved in glucose and lipid metabolic disorders, there remains a lack of understanding of the mechanism of this connection. C-reactive protein (hs-CRP) is a generic marker for inflammation that is increasingly being used clinically. A direct or indirect interaction between CRP and thyroid hormones or anti-thyroid antibodies has been suggested but not proven. A potential interaction leading to weight gain remains to be investigated. There are conflicting data on how hs-CRP may be affected by thyroid hormones in particular to thyroiditis. Interestingly, elevated hs-CRP levels were shown to be related to a decrease in deiodinase activity [61]. This relationship connects to the possibility of deiodinase enzymes being potential pathways for unexpected weight gain in post-thyroidectomy patients. Moreover, hs-CRP is correlated with obesity, insulin resistance, metabolic syndrome, and adiposity [62,63]. While one study showed no effect on hs-CRP levels in women experiencing euthyroidism during levothyroxine treatment for subclinical hypothyroidism, research must further study whether hs-CRP is affected by total thyroidectomy [64,65,66]. If so, hs-CRP’s influence on deiodinase activity and patient metabolism must also be considered. Another study observed elevated hs-CRP levels in non-obese AITD patients with TPOAb as an independent influencing factor of hs-CRP. Non-obese AITD patients with euthyroidism were shown to have higher levels of hs-CRP and TPOAb, suggesting that a chronic inflammatory state may contribute to increased insulin resistance [67]. Further research should consider whether hs-CRP and TPOAb have a long-term influence on weight gain for post-thyroidectomy non-obese patients.

Post-thyroidectomy patients who experience unexpected weight gain may also experience various levels of thyroid antibody levels. In comparison to how their levels were pre-procedure, studies have shown that TgAb levels in papillary thyroid carcinoma (PTC) patients tend to increase after thyroidectomy or radioiodine treatment due to the body’s natural immune response to intervention [68,69]. For Hashimoto’s thyroiditis patients who underwent thyroidectomy, titers of TPOAb would decrease over five years after surgery on levothyroxine treatment. Some of these patients may still exhibit TPOAb titers at an abnormal range [59]. These elevated levels of thyroid antibodies post-thyroidectomy could potentially influence a patient’s metabolism. For example, TPOAb is associated with a higher estradiol-to-testosterone ratio, an interesting relationship considering hypothyroidism’s connection with obesity, visceral fat accumulation, and high TSH values in postmenopausal women [70,71].

The interaction between inflammatory markers, thyroid antibodies, and thyroid hormones must be further investigated to better understand whether they have any potential influence on each other in influencing weight gain for post-thyroidectomy patients.

## 8. Patient-Centered Management

While physicians have practiced under the guidelines of TSH-targeted levothyroxine therapy for years, the challenge of administering hormonal therapy to replicate its endogenous secretion while also understanding the patient’s physiology remains a challenge and a “puzzle”. Therefore, the call for individualized therapy is beginning to rise [17]. Recently, physicians have been prescribing combination therapy of levothyroxine with liothyronine though there are not enough data to support the effectiveness of liothyronine use. Given the topics discussed, serum T3 could be a better marker of both treatment effectiveness and energy expenditure in thyroidectomy and levothyroxine-treated patients. A strong understanding of a patient’s physiology pre- and post-procedure/treatment through these serum levels could further deepen how a physician would continue care. In addition, the rise of the genomic medicine paradigm opens a new venue for individualized care once T3 mediators are better understood. Lifestyle monitoring, such as pre- and post-diet regulations, may also have been considered. Moreover, additional strategies such as lifestyle interventions, psychological support, and individualized hormone monitoring could provide a more holistic approach to patient management. These patient-centered care options have the potential to serve as breakthrough methods in managing patients who suffer from post-thyroidectomy metabolic consequences. 

## 9. Conclusions

To further our understanding of the metabolic consequences of thyroidectomy, a patient-centered approach must be prioritized. As such, the field must redefine what is considered “normal” and how it is reflected in thyroid hormone values. What is considered physiologically normal for a patient may not necessarily abide by the current standardized values of TSH and T4. The influence of levothyroxine and thyroidectomy on deiodinase conversions must also be a priority in future research. Mediators of T3, such as targeted physiological systems, genetic predisposition, and signaling pathways, are the most unknown concerning the metabolic consequences of thyroidectomy. Inflammatory markers, thyroid antibodies, and their potential effects on metabolism should also be considered. Overall, a patient-centered approach must be prioritized by physicians to properly treat and care for post-thyroidectomy patients as there is simply not one standard that could be applicable to all.
